# The Role of NF-κB in Intracranial Aneurysm Pathogenesis: A Systematic Review

**DOI:** 10.3390/ijms241814218

**Published:** 2023-09-18

**Authors:** Dilaware Khan, Jan Frederick Cornelius, Sajjad Muhammad

**Affiliations:** 1 Department of Neurosurgery, Medical Faculty and University Hospital Düsseldorf, Heinrich-Heine-Universität Düsseldorf, 40225 Düsseldorf, Germany; dilaware.khan@med.uni-duesseldorf.de (D.K.);; 2 Department of Neurosurgery, University Hospital Helsinki, Topeliuksenkatu 5, 00260 Helsinki, Finland; 3 Department of Neurosurgery, King Edward Medical University, Lahore 54000, Pakistan

**Keywords:** intracranial aneurysm, cerebral aneurysm rupture, NF-κB, inflammation, matrix metalloproteinases, animal models, pharmacological treatments

## Abstract

Intracranial aneurysms (IAs) are abnormal dilations of the cerebral vessels, which pose a persistent threat of cerebral hemorrhage. Inflammation is known to contribute to IA development. The nuclear factor “kappa-light-chain-enhancer” of activated B-cells (NF-κB) is the major driver of inflammation. It increases the expression of inflammatory markers and matrix metalloproteinases (MMPs), which contribute heavily to the pathogenesis of IAs. NF-κB activation has been linked to IA rupture and resulting subarachnoid hemorrhage. Moreover, NF-κB activation can result in endothelial dysfunction, smooth muscle cell phenotypic switching, and infiltration of inflammatory cells in the arterial wall, which subsequently leads to the initiation and progression of IAs and consequently results in rupture. After a systematic search, abstract screening, and full-text screening, 30 research articles were included in the review. In this systematic review, we summarized the scientific literature reporting findings on NF-κB’s role in the pathogenesis of IAs. In conclusion, the activation of the NF-κB pathway was associated with IA formation, progression, and rupture.

## 1. Introduction

Intracranial aneurysms are asymptomatic abnormal dilations of the intracranial blood vessels with an increased diameter compared to the parent artery. IAs occur in 3-5% of the population with a gender ratio of 1:1 and a mean age of 50, but after the age of 50 years, the gender ratio changes significantly with the increasing number of female cases [1,2]. The rupture of IAs is a persistent risk for hemorrhage with an incidence rate of around 7.9 per 100,000 person-years [3]. About 15% of intracranial hemorrhage patients die before reaching a hospital. Even after state-of-the-art medical intervention, the mortality rate of SAH patients is very high (~40%) [4,5], and the rupture of IAs can lead to a lifelong disability.

The pathophysiology of IAs is complex. Although many factors play together in the formation, progression, and rupture of IAs, the research hitherto suggests that inflammation heavily contributes to IAs from formation to rupture [6,7]. The endothelial dysfunction, smooth muscle cells (SMCs) phenotypic switching, infiltration and accumulation of inflammatory cells in the arterial walls, and the expression and release of pro-inflammatory cytokines such as interleukin (IL) -1β, and tumor necrosis factor-alpha (TNF-α), chemokines such as monocyte chemoattractant protein-1 (MCP-1), and IL-8, cell adhesion molecules, namely, vascular cell adhesion molecule 1 (VCAM-1) and intercellular adhesion molecule-1 (ICAM-1), and extracellular matrix remodeling proteinases such as MMPs including MMP-2 and MMP-9 have been implicated in IA formation and rupture [6,7]. The expression of these pro-inflammatory markers and MMPs is regulated by NF-κB transcription activity [7]. Experimental studies have shown that NF-κB activation increases the expression of these markers, and by blocking NF-κB activation, the expression of these inflammatory markers and MMPs could be reduced [8,9,10]. Previously, deficiency of NF-κB subunit P50 in mice has been shown to decrease the incidence of IA formation and reduced macrophage infiltration [8]. Moreover, silencing the adenomatous polyposis coli (APC) gene in rats promoted IA formation with increased NF-κB protein expression and activation [11]. It is worth noting that potential acquired risk factors for IA formation and rupture including smoking, alcohol abuse, obesity, and oxidative stress activate NF-κB and increase the expression of pro-inflammatory markers and MMPs [12,13,14,15].

In this systematic review, we focused on the contribution of NF-κB in the formation, progression, and rupture of IAs.

## 2. Methods

### 2.1. Systematic Literature Search

The PRISMA guidelines were followed for the scientific literature search. The search for research articles reporting findings on NF-κB’s role in IA formation, progression, and rupture was conducted in June 2023 in PubMed, BASE, and Embase search engines. The detailed search strategy was “NF-κB” or “NF-kappaB” in the “intracranial aneurysm” and “cerebral aneurysms” and “ruptured cerebral aneurysm” and “intracranial aneurysm” and “Un-ruptured intracranial aneurysm” and “Unruptured intracranial aneurysm” and “Un-ruptured cerebral aneurysms” and “Unruptured cerebral aneurysms”. The search results were deduplicated, and the irrelevant research articles were screened out after reading the titles.

### 2.2. Literature Screening Criteria

The original peer-reviewed research article reporting the findings, which included expression of NF-κB in IA walls in animal or human studies, targeting gene manipulation to block NF-κB activation, and using pharmacological compounds or other strategies that directly or indirectly manipulated NF-κB expression and/or activation were included in this review. The studies reporting NF-κB findings in human IAs with only mutated genomes without comparing them to normal genomes were excluded. 

## 3. Results

**Figure 1:**
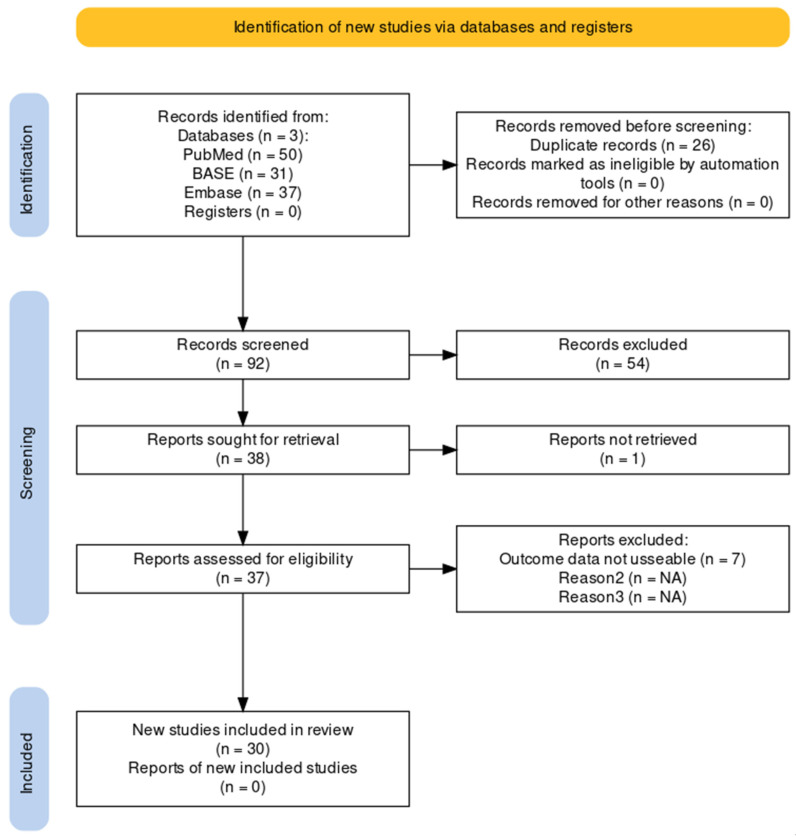
Flowchart showing a systematic search of the literature [16]. A systematic search for scientific literature was performed in June 2023.

The systematic search on search engines PubMed, Embase, and BASE for scientific literature reporting NF-κB’s role in aneurysm biology delivered a total of 118 records. After deduplication and title screening, 92 unique research articles were left. The abstract screening delivered 38 research articles for full-text screening. The full-text screening delivered 30 scientific research articles. The flowchart for the systematic search is shown in Figure 1. Eight research articles reported findings on human samples, and 25 studies reported NF-κB findings using animal models. Three studies reported both experimental and clinical data. 

### 3.1. Clinical Studies

Seven clinical studies reported NF-κB expression and/or activation in IAs compared to normal cerebral or nonvascular diseased arteries. The findings of these studies are summarized in Table 1. One study provided findings on NF-κB genotype correlation to IA formation. According to this study, ATTG1/ATTG2 and ATTG2/ATTG2 genotypes compared with the ATTG1/ATTG1 genotype were linked to a significantly decreased risk of IAs, suggesting that the ATTG2 allele may be a protective factor against IA formation [17].

### 3.2. Animal Experimental Studies

Among the included studies, 16 studies reported findings on NF-κB’s role in IA formation, progression, and rupture. In addition to NF-κB, these studies reported findings on the regulation of inflammatory markers, MMPs, internal elastic lamina (IEL) loss, media thinning, vascular smooth muscle cells (VSMCs) loss and apoptosis, and macrophage infiltration. The findings of these studies are summarized in Table 2. Three studies used rabbits, 18 studies used rats, and 8 studies used mouse models of IAs. In rabbit models, the common carotid artery was ligated to induce IAs. In rats, all studies used left common carotid artery and bilateral posterior renal arteries ligation with increased salt intake with or without β-aminopropionitrile (BAPN) to induce IAs. In mice, IAs were induced either by using the same IA model as that of rats or by using deoxycorticosterone acetate-salt hypertension combined with a single elastase injection with or without BAPN.

In total, 21 studies used different approaches to decrease the incidence of aneurysm formation and rupture and to ameliorate aneurysmal changes. Among these studies, 2 studies used the rabbit IA model, 6 studies used the rat IA model, and 7 studies used the mouse model of IAs. In all studies, the NF-κB expression and/or activation was directly or indirectly inhibited, except in one study, where the protein expression and phosphorylation of NF-κB P65 were enhanced. The findings of these studies are summarized in Table 3.

## 4. Discussion

The clinical studies provided ample convincing evidence that the NF-κB P65 subunit is overexpressed at mRNA and protein levels with increased phosphorylation in IAs compared to normal arteries and nonvascular diseased tissue (Table 1) [8,11,19,20,21,22]. Elevated NF-κB activation was linked to the increased diameter of IAs [11]. In addition to that, polymorphism in the genotype of the NF-κB promoter was linked to a lower risk of IAs [17]. Similar to clinical studies, experimental studies using different animal models showed the elevated mRNA expression, protein levels, and/or activation of NF-κB in IA tissue compared to normal control arteries (Table 2) [8,20,23,25,26,27,29,30,31,32,33,34,40,43]. The animal experimental studies revealed that blocking NF-κB P50 expression and NF-κB activation reduced the incidence of IA formation (Table 3) [8,40], while enhanced NF-κB activation was linked to increased IA formation (Table 3) [11]. Moreover, the treatments, which reduced the formation, growth, and rupture of IAs, also mitigated the mRNA expression, protein levels, and/or phosphorylation of NF-κB P65 (Table 3) [20,23,25,26,29,30,31,32,33,35,36,37,38,39,40,42,43], suggesting the heavy contribution of NF-κB activation in IA formation and rupture. 

NF-κB activation increases the transcription and protein expression of inflammatory markers and MMPs [7]. Higher mRNA expression, protein levels, and concentrations of pro-inflammatory markers in serum and IA walls have been reported in clinical and experimental animal studies (Table 1 and Table 2) [11,14,20,22,23,25,27,28,29,30,31,36,43]. The mRNA and protein expression of these inflammatory markers were lowered by pharmacological treatments and genetic manipulations, which also reduced the incidence of IA formation, growth, and rupture (Table 3) [8,14,23,25,30,31,32,35,36,37,38,39,43]. Furthermore, blocking NF-κB expression and activation reduced the expression of pro-inflammatory markers including IL-1β, MCP-1, and VCAM-1 (Table 3) [8]. In addition to that, animal experimental studies showed that blocking the expression of these pro-inflammatory markers, including TNF-α, IL-1β, and MCP-1, or blocking their function via inhibiting their receptors or knocking out their receptors in mice could significantly reduce the formation, progression, and rupture of IAs [28,36,44,45]. It is interesting to note that APC gene silencing in rats, which promoted IA formation and rupture, resulted in enhanced mRNA expression, protein levels, and phosphorylation of NF-κB P65 with elevated mRNA and protein expression of TNF-α, IL-1β, IL-6, MCP-1, MMP2, and MMP-9 (Table 3) [11]. These findings demonstrate the causative role of NF-κB activation in IA pathology.

Furthermore, inhibition of NF-κB activation resulted in reduced expression of MMP-2 and MMP-9 in IA walls of experimental animals (Table 3) [8]. Higher mRNA expression, protein levels, serum concentration, and strong staining intensity of these MMPs in human and animal IA tissue have been reported (Table 1 and Table 2) [14,19,20,27,28,29,30,31,33]. Experimental studies have shown that NF-κB activation results in increased transcription and protein levels of both MMPs [8,9,10]. The strategies, which lowered the incidence of IA formation and rupture, also reduced the mRNA expression, protein levels, and/or activation of MMPs (Table 3) [8,14,20,23,24,25,28,29,30,31,33,35,38,39,42]. Moreover, blocking the expression and activation of these MMPs reduced the formation and progression of IAs in experimental animal studies [24,46,47]. The rupture and progression of IAs depend on the balance between the proteins causing degeneration and regeneration of the extracellular matrix. NF-κB plays an important role in regulating this balance, as, on one hand, NF-κB transcription activity increases the expression of MMPs [8,9,10], and, on the other hand, it reduces the expression of procollagens and LOX [41], which can consequently destabilize IA walls, leading to IA rupture. 

NF-κB activation was detected in macrophages, endothelial cells, and SMCs in IA walls of experimental animals [8,38,40]. Macrophages were predominantly infiltrated and accumulated in IA walls compared to normal arteries in human and arteries in SHAM-operated animals, and their number increased with the progression of IAs in experimental animal studies (Table 2) [14,23,25,28,29]. In addition to macrophages, infiltration and accumulation of neutrophils, mast cells, and T cells were also detected in IA walls (Table 2) [23,35]. Blocking NF-κB expression and activation and the treatments reducing IA formation and rupture attenuated the infiltration and accumulation of these inflammatory cell types in IA tissue (Table 3) [8,23,25,28,29,32,33,35,36,37,38,39,40]. Blocking or suppressing cell-specific NF-κB activation provided interesting results. It has been shown that macrophage-specific NF-κB inhibition and suppression could potentially reduce the incidence of IA formation, while blocking and suppressing NF-κB activation in endothelial cells did not reduce the incidence of IA formation [40]. The inhibition of NF-κB in macrophages reduced CCL2 expression and, consequently, ameliorated macrophage infiltration in IA walls [40]. Contrary to these findings, macrophages do not seem to be the only culprit, as clodronate liposomes lowered the number of circulating and infiltrated macrophages in IA tissue, but it did not reduce aneurysmal damage in the rabbit model of IAs [24]. The treatment with clodronate liposomes did not reduce the mRNA and protein levels of MMP-2 and MMP-9 in IA walls [24]. Furthermore, immunofluorescence staining showed that SMCs were the source of increased MMP-2 and MMP-9 with increased NF-κB P65 and MCP-1 levels [24], suggesting the contribution of NF-κB expression and activation in SMCs to IA initiation. The vascular and inflammatory cells orchestrate the cellular and molecular events leading to IA initiation, progression, and rupture, thus development of IAs cannot be attributed to a single cell type. Moreover, different experimental animal models and time points of investigation after IA induction could affect the outcomes of the studies, which can explain the contradictory results discussed above.

Taken together, these studies suggest that the pharmacological drugs, which can block NF-κB activation and can suppress the consequent expression and release of cytokines, chemokines, cell adhesion molecules, and MMPs in macrophages and SMCs, can be suitable candidates that might reduce IA formation and rupture. Experimental animal studies will be needed to further explore the potential of these drug candidates.

## 5. Conclusions

Clinical and experimental studies have shown the activation of NF-κB in IA formation. NF-κB contributes to IA formation and rupture probably via increasing the transcription of inflammatory markers and MMPs. Blocking NF-κB expression and/or activation attenuated mRNA expression and protein levels of inflammatory markers and MMPs and reduced IA formation and rupture in different animal models. Limitations: For this systematic review, a meta-analysis was not performed. Moreover, the inclusion criteria were strictly limited to NF-κB regulation, and the studies reporting findings on NF-κB upstream/downstream signaling molecules without investigating NF-κB regulation were not included in the study.

## Figures and Tables

**Table 1:** Studies reporting NF-κB status in patients with IAs compared to control with nonvascular diseased arteries or normal cerebral arteries.

No.	NF-κB Status	Inflammatory Markers and MMPs	Ref.
1	Lower nuclear factor of kappa light polypeptide gene enhancer in B-cells inhibitor, alpha (IκB-α) and higher NF-κB P65 protein expression in IA tissue	Higher serum levels of MCP-1, TNF-α, IL-1β, and IL-6 in IA patients than in control, the serum levels of MCP-1, TNF-α, IL-1β, and IL-6 were higher in patients with ruptured CAs than in patients with unruptured IAs, lower APC protein expression in IA tissue	[11]
2	Lower median cerebrospinal fluid (CSF) and serum NF-κB p65 concentrations	Higher median CSF GRO alpha chemokine/C-X-C motif ligand 1 (CXCL1) (GRO-α) and CSF C-X-C Motif Chemokine Receptor 2 (CXCR2) concentration	[18]
3	Higher NF-κB mRNA and protein expression in IAs than in control, NF-κB protein expression was detected in the intima, media, and extima	Higher MMP-2 mRNA and protein expression in IAs than in control, MMP-2 protein expression was detected in the intima, media, and extima	[19]
4	High positive protein expression of NF-κB P65 in IA walls detected by immunohistochemical staining	Higher levels of IL-17, interferon-gamma (IFN-γ), IL-17a, MCP-1, TNF-α, IL-6, and lower levels of IL-10 in peripheral blood, high positive protein expression of phosphoinositide-3-kinase (PI3K) and protein kinase B (Akt) detected by immunohistochemical staining, high mRNA expression of T helper 17 cells (Th17) transcription-factor-related orphan receptor C (RORC) and low mRNA expression of regulatory T-cell (Treg) transcription factor forkhead box p3 (Foxp3)	[20]
5	In serum higher NF-κB 65 concentration	In serum higher mRNA expression and concentration of toll-like receptor (TLR) 2, TLR 4, and myeloid differentiation primary response 88 (MyD88)	[21]
6	Increased immunohistostaining for NF-κB P65 in the aneurysmal wall		[8]
7	Increased protein levels and staining intensity for NF-κB P65 in IA tissue	Higher protein levels and increased staining intensity for ICAM-1 and higher MCP-1 mRNA expression in IA tissue	[22]

**Table 2:** Animal studies reporting NF-κB status in IA animals compared to control animals.

	Animals	NF-κB Activation	Inflammatory Markers and MMPs	IA Features	Ref.
1	Rats	Higher NF-κB P65 mRNA expression, increased protein levels of NF-κB p-p65	Higher mRNA levels of TLR4, Poly (ADP-ribose) polymerase-1 (PARP-1), TNF-α, inducible nitric oxide synthase (iNOS), MMP-2, and MMP-9, increased protein expression of TLR4 and PARP-1	Stratification in the cerebral artery wall, decreased SMCs, inward depressing exited in the vascular wall, infiltration, and accumulation of macrophages, neutrophils, and T cells	[23]
2	Rabbits	Increased NF-κB-p65 staining intensity	MMP-2 and MMP-9 in SMCs, increased staining intensity of MMP-2, MMP-9, and MCP-1, lower smooth muscle actin (SMA) and calponin	IEL loss, media thinning, and bulge formation within one week, larger zones of media thinning and bulging 6 months later	[24]
3	Rats	Higher mRNA and protein expression of NF-κB P65 and lower mRNA and protein expression of IκBα, increased NF-κB p65 phosphorylation	Higher mRNA protein expression of MMP-2, MMP-9, TNF-α, IL-1β, Il-6, and lower mRNA and protein expression of APC	Damaged endothelium, degenerated VSMCs, lower number of VSMCs and its layers, thinner artery wall, fractured elastic fiber, and inflammatory cell infiltration	[11]
4	Rats	Higher mRNA expression of NF-κB in IA walls	Higher mRNA expression of MMP-2, MMP-9, MCP-1, and VCAM-1 in IA walls	Increased macrophage infiltration in IA walls	[25]
5	Rats	Higher levels of phosphorylated NF-κB P65 and IκBα, lower levels of IκBα protein	Decreased tumor necrosis alpha-induced protein 3 (A20) protein expression	Disrupted IEL	[26]
6	Mice	mRNA expression of NF-κB increased in unruptured IAs and even more in ruptured IAs	Myocardin, smooth muscle alpha-actin (SM-α-actin), smooth muscle myosin heavy chain (SM-MHC), and SM-22α mRNA levels decreased, while MCP-1, MMP-3, MMP-9, TNF-α, IL-1β, iNOS, VCAM, and Krüppel-like factor 4 (KLF4) increased in unruptured IAs and even more in ruptured IAs	Layers of discontinuous endothelial cells and scattered VSMCs, disorganized elastic lamina, macrophage infiltration, and NADPH oxidase-1 (NOX1) immunoreactivity was significantly higher in unruptured IAs, and highest in ruptured IAs, colocalizing with both SMCs and macrophages	[14]
7	Rabbits	Increased the protein expression of phosphorylated inhibitory-κB kinase alpha (p-IKKα) and t-IKKα and positive expression rate of NF-κB P65	Decreased eNOS mRNA expression and increased iNOS mRNA expression. Staining intensity and mRNA expression of MMP-2 and MMP-9 increased. The expression of Th17-related factors RORYT, IL-17, IL-22, IL-23, and RORC were increased, and the expression of Treg-related factors IL-10, TGF-β, and Foxp3 was decreased, increased protein expression of t-PI3K, p-PI3K, t-AKT, p-AKT.	Increased length of IEL loss and media thinning, reduced SMCs, broken elastic fibers, staining intensity, and mRNA expression of α-SMA was decreased. The number of Th17 cells was increased and the number of Treg cells was decreased in IA walls.	[20]
8	Rabbits	The mRNA and protein expression of NF-κB peaked one week after IA induction.	The mRNA and protein expression of MCP-1 peaked one week after IA induction. MMP-9 protein expression increased gradually.	Fractured elastic fiber, lower number of SMCs, damaged endothelial cells	[27]
9	Rats	NF-κB p65 expression colocalized with MCP-1	MCP-1 expressed in intima, media, and adventitia, localized to IA walls, increase in MCP-1 protein expression with IA progression	Macrophage accumulation in IA walls increased with IA progression.	[28]
10	Rats	Increased mRNA expression of NF-κB	Increased mRNA expression of MMP-2, MMP-9, VCAM-1, MCP-1 and decreased mRNA expression of eNOS and (issue inhibitor matrix metalloproteinase 1 (TIMP-1)	Increased macrophage infiltration and increased SMC apoptosis, decreased mRNA expression of B-cell lymphoma 2 (Bcl-2), and increased mRNA expression of iNOS	[29]
11	Rats	Increased staining intensity for NF-κB P65, NF-κB was activated in both endothelial cells and macrophages	MCP-1 and VCAM-1 costained with NF-κB P65		[8]
12	Mice	Increased protein expression of janus kinase 2 (JAK2), signal transducer and activator of transcription 3 (STAT3), and NF-κB P65, increased phosphorylation of STAT3 and NF-κB P65	Increased relative mRNA expression and release of TNF-α, IL-1β, IL-6, MCP-1, and IFN-γ and reduced IL-10 Reduced mRNA expression of MHC, SMA, and SM22 and increased mRNA expression of MMP-2 and MMP-9		[30]
13	Rats	increased mRNA expression of NF-κB	Increased concentration of IL-1β, IL-2, IL-6, IL-8, IL-17, and TNF-α, and increased MMP-2 and MMP-9 levels in IA walls, increased IFN-γ and SM22, increased NAD(P)H quinone dehydrogenase 1 (NQO-1) levels Decreased cytoplasmic nuclear factor erythroid-2-related factor (Nrf)-2 and increased nuclear Nrf-2	Increased macrophage infiltration and increased reactive oxygen species (ROS)	[31]
14	Rats	Increased DNA binding activities of NF-κB	Increased DNA binding activities of protein C-ets-1 (Ets-1)	Disrupted IEL and media thinning	[32]
15	Mice	Increased mRNA and protein levels of NF-κB	Increased mRNA and protein expression of MMP-2 and MMP-9	Decreased thickness of the arterial wall Increased macrophage infiltration	[33]
16	Rats	Increased protein expression and phosphorylation of NF-κB P65	Expression of TLR10 mRNA gradually increased with cerebral aneurysm progression. mRNA, protein expression, and staining intensity of TLR-4 increased in IA walls after one month and decreased after three months. Expression of TLR4 coincided well with NF-κB P65 activation.		[34]

**Table 3:** Studies reporting the effects of pharmacological/other treatments or genetic manipulations on NF-κB status and IA formation/rupture in animals.

No.	Animals	Treatments	Genetic Manipulation	NF-κB Status and Other Pathways	Inflammatory Markers and MMPs	IA Features	Ref.
1	Rats	3-amino benzamide (3-AB)		Reduced NF-κB P65 mRNA expression and decreased protein levels of NF-κB p-p65	Suppressed mRNA expression of TLR-4, PARP-1, TNF-α, iNOS, MMP-9, and MMP-2 and reduced protein expression of TLR-4, PARP-1	IA formation 66.7% in control vs. 54.8% in 3-AB, decreased cerebral artery wall damage, weaker inward depressing, reduced accumulation of macrophages, neutrophils, and T cells	[23]
2	Rat		APC-siRNA	Enhanced mRNA and protein expression of NF-κB P65 and inhibited mRNA and protein expression of IκBα, increased NF-κB p65 phosphorylation	Enhanced mRNA and protein expression of MMP-2, MMP-9, TNF-α, IL-1β, Il-6, and inhibited mRNA and protein expression of APC	Endothelium disappeared, worsened VSMC degeneration, lowered the number of VSMCs and its layers, with the thinnest artery wall, severely fractured elastic fiber, and increased inflammatory cell infiltration	[11]
3	Rats	Aspirin		Lowered mRNA expression of NF-κB in IA walls	Lowered the mRNA expression of MMP-2, MMP-9, MCP-1, and VCAM-1 in IA walls	Smaller aneurysm size with reduced macrophage infiltration, IEL score, and media thinning	[25]
4	Rats	ZnSO_4_		Reduced phosphorylation of NF-κB p65 and IκBα in IA walls	Increased A20 expression in IA walls	Prevented IA growth, smaller IA size, increased wall thickness ratio, suppressed macrophage infiltration	[26]
5a	Mice	Apocynin		Decreased mRNA expression of NF-κB	Decreased mRNA expression of MCP-1, MMP-3, MMP-9, TNF-α, IL-1β, iNOS, VCAM, and KLF4 Increased myocardin, SM-α-actin, SM-MHC, and SM-22α mRNA levels	IA formation 84% in control vs. 32% in apocynin, aneurysm rupture 60% in control vs. 12% in apocynin	[14]
5b	Mice		p47phox−/−	Decreased mRNA expression of NF-κB	Decreased mRNA expression of MCP-1, MMP-3, MMP-9, TNF-α, IL-1β, iNOS, VCAM, and KLF4 Increased myocardin, SM-α-actin, SM-MHC, and SM-22α mRNA levels	IA formation 84% in control vs. 16.7% in p47phox−/−, IA rupture 60% in control vs. 8.3% in p47phox−/−	[14]
6	Rat	Tranilast		Inhibited the protein expression of NF-κB p65 in IA walls	Reduced mRNA expression and inhibited protein expression of IL-1 beta, MCP-1, MMP-2, and MMP-9 in IA walls	Suppressed the size of the induced IAs and thinning of the media, prevented the disruption of the IEL and the degeneration of the media, lowered the number of infiltrated macrophages	[35]
7	Mice	N/A	Tumor necrosis factor receptor superfamily (TNFR)−/−	Suppressed NF-κB activation in IA lesions	Suppressed mRNA and protein expression of MCP-1 and cyclooxygenase-2 (COX-2) in IA lesions	IA formation 68% in control vs. 12% in TNFR−/−. Inhibited macrophage infiltration in IA lesions	[36]
8	Rats	Anagliptin		Reduced NF-κB P65 phosphorylation in macrophages in IA walls	Reduced MCP-1 protein expression in IA walls and lowered MCP-1 mRNA expression in cerebral arteries	Suppressed IA growth, decreased size of IEL disruption and IAs. Increased wall thickness ratio, decreased lumen area of aneurysm, less macrophage infiltration into the IA walls	[37]
9	Rabbits	Bone marrow mesenchymal stem cells (BMSCs) exosomes		Reduced protein expression of p-IKK-a, t-IKK-a and decreased positive expression rate of NF-κB P65	Increased eNOS mRNA expression and decreased iNOS mRNA expression Reduced mRNA expression and staining intensity of MMP-2 and MMP-9 Decreased the expression of Th17-related factors RORYT, IL-17, IL-22, IL-23, and RORC Increased the expression of Treg-related factors IL-10, TGF-β, and Foxp3, reduced protein expression of t-PI3K, p-PI3K, t-AKT, p-AKT	Reduced length of IEL loss and media thinning, staining intensity, and mRNA expression of α-SMA were increased Decreased T17 cells and increased T-regs in IA walls	[20]
10	Mice	N/A	MCP-1−/−	Did not affect the number of cells expressing NF-κB P65 in IA walls	Reduced staining intensity and mRNA expression of MMP-2, MMP-9, and iNOS in IA walls	Reduced aneurysmal changes, decreased IEL disruption, reduced macrophage infiltration	[28]
11	Rats	Endothelial colony-forming cells (ECFCs) transfusion		Decreased mRNA expression of NF-κB	Decreased mRNA expression of MMP-2, MMP-9, VCAM-1, and MCP-1, increased mRNA expression of eNOS, TIMP-1, and Bcl-2, and decreased mRNA expression of iNOS	Decreased aneurysm size and SMC apoptosis Increased media thickness and inhibited macrophage infiltration	[29]
12a	Mice	N/A	p50−/−		Inhibited elevation in MCP-1, VCAM-1, MMP-2, MMP-9, IL-1β, and iNOS mRNA expression	Aneurysm formation 70% in p50+/+ vs. 10% in p50−/−. Reduced IEL disruption, smaller aneurysm size, reduced macrophage accumulation	[8]
12b	Rat	NF-κB decoy ODN			Lower mRNA expression of MCP-1, VCAM-1 MMP-2, MMP-9, IL-1, and iNOS. Reduced staining intensity for MCP-1 and VCAM-1	Aneurysmal changes 100% in control vs. 40% in NF-κB decoy ODN, Reduced IEL disruption, smaller aneurysm size, reduced macrophage infiltration	[8]
13	Rats	Nifedipine		Decreased staining intensity for NF-κB P65 in CA walls. Reduced DNA binding of NF-κB P65 in IA walls	Reduced staining intensity and mRNA expression of MCP-1 and MMP2	Reduced aneurysm size No effect on IEL Increased thickness of media Decreased macrophage infiltration	[38]
14a	Mice		Prostaglandin E receptor 2 (Ptger2)−/−	Suppressed NF-κB activation and staining intensity for phosphorylated NF-κB P65	Suppressed COX-2 expression in CA walls Suppressed protein expression of iNOS and MMP2 Reduced staining intensity for MCP-1, IL-1beta, iNOS, and MMP2	IA formation is almost absent, reduced IEL disruption, reduced macrophage infiltration in IA walls	[39]
14b	Rats	celecoxib		Suppressed NF-κB activation	Suppressed EP2 expression in CA walls. Reduced MMP-2, MCP-1 and IL-1β expression	Reduced IA formation, decreased IA size, reduced macrophage infiltration in IA walls	[39]
15	Mice	BP-1-102		Reduced the protein expression of JAK2, NF-κB P65, and STAT3 Decreased the phosphorylation of NF-κB P65 and STAT3	Increased mRNA expression of SMCs markers MHC, SMA, and SM22 Reduced mRNA expression of MMP-2 and MMP-9 and reduced the mRNA expression and release of TNF-α, IL-1β, IL-6, MCP-1, and IFN-γ Increased the mRNA expression and release of IL-10	IA rupture 81% in control vs. 37% in BP-1-102	[30]
16a	Mice		Ptger2f/ fLyz2Cre	Suppressed NF-κB activation		Less IA formation and suppressed macrophage recruitment in IA walls	[40]
16b	Mice		Ptger2f/ fCdh5Cre	Did not suppress NF-κB activation		Did not affect IA formation and macrophage infiltration	[40]
16c	Mice		IkB mutant– Lyz2Cre	Reduced staining intensity for NF-κB p-P65 in IA walls	The mRNA expression of MCP-1 and Ptger2 was abolished.	Less IA formation and reduced macrophage infiltration	[40]
16d	Mice		IkB mutant–Cdh5Cre		Did not affect mRNA expression Ptger2	Did not affect IA formation and macrophage infiltration	[40]
16e	Mice	F-04418948		Reduced phosphorylation of NF-κB P65	Reduced staining intensity for COX-2 and MCP-1	Decreased the size of IAs, reduced thinning of media, suppressed macrophage infiltration	[40]
17	Rats	PCA	N/A	Reduced mRNA expression and activation of NF-κB	Reduced concentration of IL-1β, IL-2, IL-6, IL-8, IL-17, and TNF-α and reduced MMP-2 and MMP-9 levels in CA walls Decreased IFN-γ and SM22 and increased NQO-1 levels Decreased cytoplasmic Nrf-2 and increased nuclear Nrf-2	Reduced aneurysm size, decreased tunica-media thickness, reduced macrophage infiltration, increased ROS	[31]
18	Rats	NF-κB Decoy ODN	N/A			Higher mRNA expression of procollagen 1(I), 1(III), and lysyl oxidase (LOX) in IAs	[41]
19a	Rats	NF-κB decoy ODN			Inhibited the up-regulated mRNA expression and staining intensity of MCP-1 mRNA in CA walls Partially restored the decreased mRNA expression of procollagen α1 and increased staining intensity for procollagen α1	Suppressed IEL disruption and prevented media thinning Did not restore medial thickness, suppressed IA enlargement, did not reduce the size of preexisting IAs, inhibited macrophage infiltration	[32]
19b	Rats	Chimeric decoy ODN		Inhibited DNA binding activities of both NF-κB and Ets-1	Inhibited the up-regulated expression of MCP-1 mRNA and suppressed staining intensity for MCP-1 in IA walls Restored the mRNA expression of procollagen α1 (type I and type III), increased staining intensity for procollagen α1 (type I and type III)	Suppressed IEL disruption, prevented media thinning, increased medial thickness, suppressed the IA enlargement, diminished the size of preexisting IAs, inhibited macrophage infiltration	[32]
20	Mice	Resveratrol	N/A	Reduced mRNA and protein level of NF-κB	Reduced mRNA and protein levels of MMP-2 and MMP-9.	IA formation 66.7% in control vs. 16.6% in resveratrol Reduced IA size and thicker arterial wall Reduced macrophage infiltration	[33]
21	Rats	Tan IIA	N/A	Reduced NF-κB mRNA expression and activation in IAs	Reduced mRNA expression of MCP-1, MMP-2, and MMP-9	Suppressed IA growth, decreased IA size, increased arterial wall thickness, less macrophage infiltration	[42]
22	Rats	N/A	shTWIST1	Decreased phosphorylation of NF-κB P65 and IκB Increased IκB protein expression	Increased expression of Bcl-2 and decreased expression of Bax and cleaved caspase-3 Reduced serum levels of TNF-α and IL-6	Ameliorated vessel tissue degeneration in IA walls, suppressed VSMC apoptosis	[43]
